# Lifestyle score and oral health behavior impact on oral health-related quality of life among nursing students, Ranchi, Jharkhand, India

**DOI:** 10.6026/973206300220903

**Published:** 2026-02-28

**Authors:** Sandeep Kumar, Arunoday Kumar, Amit V. Mahuli, Sudhadevi M, Jay Kishore, Debashish Basak, Punit Karmacharya

**Affiliations:** 1 Department of Public Health Dentistry, Dental Institute, RIMS, Ranchi, Jharkhand, India; 2 Department of Prosthodontics, Dental College, RIMS, Imphal, Manipur, India; 3 College of Nursing, RIMS, Ranchi, Jharkhand, India; 4 Department of Oral Pathology and Microbiology, Patna Dental College and Hospital, Patna, Bihar, India; 5 Department of Prosthodontics, Hazaribagh College of Dental Sciences and Hospital, Hazaribagh, Jharkhand, India; 6 Department of Dentistry, B.P. Eye Foundation, Bhaktapur, Nepal

**Keywords:** Lifestyle, oral health behavior (OHB), oral health quality of life (OHRQoL), nursing students

## Abstract

Nursing students are future healthcare providers and there is limited evidence on how lifestyle and oral health behavior influence oral health quality of life (OHRQoL) in nursing students. This cross-sectional study was conducted in the Ranchi district and included 400 nursing students to assess the lifestyle score, oral health behavior and OHRQoL using their standardized and validated tool. The data was collected using a self-administered questionnaire and showed that 75.8% of nursing students had never been to a dentist for an oral health examination and more than two-thirds of the nursing students demonstrated good Hu-DBI Scores (70.5%) and good OHIP-14 Scores (75.5%). The number of nursing students demonstrating good HPI Scores was very low (8.3%) while, the nurses who had poor Hu-DBI Scores (OR=2.057, p value=0.001) and poor HPI Scores (OR=2.174, p value=0.043) were significantly more likely to have poor OHRQoL. There is a need for policies that will improve the oral health behavior and lifestyle factors of nursing students.

## Background:

It requires a unique blend of empathy, patience and resilience, as nurses often face challenging situations that demand both emotional and physical strength. Their role is crucial in providing holistic care. The practice of nursing in India involves providing healthcare to patients, their families and their communities. A nurse's role is vital in promoting health, preventing disease and providing primary and community care. The role of these professionals is to provide emergency care and to make sure that all people have access to health coverage. Nursing students place little emphasis on oral health care, despite it being one of the most basic aspects of nursing. There is a lack of adequate oral health knowledge among qualified nursing staff and nursing students on medical wards, resulting in inadequate oral healthcare for patients [1]. As healthcare workers, nurses are directly responsible for maintaining the oral health of patients. Preparing them for their future work as public health workers requires assessing their "Oral health behavior (OHB). "The habits and practices that affect a person's oral health are called "OHB" [2, 3]. Among these are brushing your teeth, flossing, using mouthwash and going to the dentist. In addition to diet, smoking and dental anxiety, other factors impact oral health. According to a number of previously published studies, good OHB is associated with better OHRQoL [4, 5 and 6]. "Lifestyle (LS)" is another factor that is known to influence the OHRQoL. According to Backett & Davison [7], LS is "a behavior associated with an individual or group". Healthier lifestyle patterns are associated with more positive dental behaviors (e.g., better oral hygiene), supporting the link between LS and oral health behaviors that impact OHRQoL [8, 9]. There have been studies conducted linking LS and OHB with OHRQoL independently, but their joint association has rarely been investigated. Furthermore, nursing students' LS, OHB and OHRQoL have never been assessed. This study advances existing knowledge by jointly examining lifestyle and oral health behavior and their impact on oral health-related quality of life among nursing students. It provides evidence from under-researched populations in Ranchi district, Jharkhand. Therefore, it is of interest to undertake this study with the aim of assessing the impact of LS and OHB on OHRQoL in nursing students in Ranchi district, Jharkhand.

## Materials and Methods:

This study assessed the impact of LS and OHB score on OHRQoL in nursing students in the Ranchi district. The participants in this cross-sectional study were nursing students enrolled at various nursing colleges in the Ranchi district. The data collection for the study lasted for nearly one month. The participants in the study were between the ages of 18 and 25 years. According to the results of the pilot study (effect of 55% on OHRQoL, power=80%, alpha error=5%), as well as the formula suggested by the "World Health Organization (WHO) for calculating sample size", it was found that a minimum of 374 would be considered adequate [10]. The actual number 400 was rounded off. We employed a stratified simple random sampling technique for sample selection. Ranchi district was divided into four zones to collect statistics. One nursing college was chosen at random from each zone. One hundred nursing students in all were chosen from each zone. Thus, this study consisted of 400 nursing students in total. The Institutional Ethics Committee gave its approval to conduct this study. The study participants were asked for their informed consent. Included were the nursing students who signed the informed consent form and expressed their interest in taking part. Reluctant participants in the research were not included. A self-administered questionnaire collected the requisite information. Data on socio-demographic characteristics, dental visit frequency and oral hygiene habits were gathered in the first section. The Health Practice Index (HPI) was used to evaluate LS factors. "Good health practices" are coded as 1 and "Poor health practices" are coded as 0 on a 2-point scale used to evaluate the outcomes. As a result, the lowest and greatest scores fall between 0 and 8. The "lifestyle" was then separated into three categories based on Morimoto's criteria: "poor (0-3), intermediate (4-5) and good (6-8)" [11]. The Hu-DBI Index was used to evaluate oral health behavior. It has twenty yes/no responses. As the maximum score in Hu-DBI is 12, a higher score reflects better oral health practices [12]. The OHIP-14 questionnaire was used, which has a set of 14 questions with a minimum and maximum score ranging from 0 to 56. A higher score denotes a lower quality of life associated with dental health [13]. The "modified Kuppuswamy scale" was used for calculating social status, which took into consideration Income, education and occupation [14]. SPSS V 20 (SPSS Inc., Chicago, Illinois, USA) was used to examine the gathered data after it was assembled and entered into an Excel sheet. Chi-square testing and frequency distribution analysis were carried out. Regression analysis was used to assess the impact of LS choices and OHB on OHRQoL. In addition, other variables linked to poor OHRQoL were tested and the strength of association between them was assessed under 0.05, a p-value is considered statistically significant.

## Results:

The mean age of the study population was 22.10±1.84 years. Most of them used toothbrushes (83.3%) and toothpaste (87.3%) to maintain oral hygiene. Nearly two-thirds of the nursing students (67.8%) were from lower strata of society, 32.3% were from the middle class and none of them belonged to the upper class. More than two-thirds of the nursing students (75.8%) had never visited a dentist before for an oral health check-up (Table 1). More than two-thirds of the nursing students demonstrated good Hu-DBI Score (70.5%) and good OHIP-14 Score (75.5%). On the contrary, very few nursing students demonstrated good HPI Scores (8.3%) (Table 2). The OHRQoL was dichotomized at its median value into "good" and "poor". Out of the various factors tested, Hu-DBI Scores (p value=0.007) and HPI Scores (p value=0.041) showed a significant association with the OHRQoL. The other factors, like location, method of cleaning, material used for cleaning, frequency, previous dental visit and socioeconomic status, did not show any significant association (Table 3). The factors that showed a significant association with OHRQoL were then kept in a "multivariate Logistic regression model," and the adjusted Odds ratio was computed. The nurses who were having poor Hu-DBI Scores were significantly more likely to have poor OHRQoL (OR=2.057, p value=0.001). Poor HPI scores were also associated with poor OHRQoL (OR=2.174, p value=0.0430) (Table 4).

## Discussion:

A total of 400 nursing students in different nursing colleges in Ranchi participated in this cross-sectional study. According to the findings of the study, the majority of students enrolled in Ranchi's nursing colleges come from rural areas and are from lower socioeconomic backgrounds. The nursing profession offers excellent job opportunities and better placement, which is why rural residents favor it over other professions. It was found that the majority of nursing students practiced good oral hygiene. Despite of majority of them having a rural background, they were brushing their teeth using toothbrushes and toothpaste and demonstrated good oral hygiene techniques. Hence, they scored well on the Hu-DBI Index score. The authors think that nursing students are healthcare professionals who are taught about sterilization, disinfection and good practices in their nursing curriculum. A comprehensive nursing curriculum may be one of the factors responsible for the majority of the students demonstrating higher/intermediate scores on HPI and Hu-DBI Index, respectively. Nursing students reported very few dental visits in the present study. In a previous study conducted by Agarwal *et al.* (2024) and Rambabu *et al.* (2018), the authors were of the opinion that in the Indian scenario, people visit the dentist only for emergency care [15, 16]. They seldom visit the dentist for preventive care. The nursing professionals have reported good oral hygiene awareness and high Hu-DBI Scores. They might not have visited a dentist, probably because there was no need to undergo emergency care. They should be encouraged to visit dentists for routine dental checkups. In the present study, good and intermediate HPI Scores were reported by the majority of the nursing students. Health and well-being are maintained throughout life through healthy habits. "A healthy diet, quitting smoking, maintaining a healthy weight, avoiding harmful alcohol consumption and exercising regularly" are part of healthy lifestyles. It is also possible to reduce the risk of chronic diseases by living a healthy lifestyle [17, 18]. The authors think that this study was conducted among healthcare professionals who have good knowledge of health and its determinants. Hence, a higher value on the HPI Index was reflected in the nursing students. The majority of the nursing students reported good OHIP-14 Scores. This is similar to the findings reported by Josh *et al.* (2024) in their study conducted on nursing professionals in India [19]. The authors think that in the present study, the majority of the nursing students had good oral hygiene awareness and practices, due to which the majority of them had good OHIP-14 Scores. Those nursing students who have poor OHIP-14 Scores may be due to less frequency of dental visits for oral care. A majority of the nursing students were from rural backgrounds and belonged to lower strata of society. Hence, financial constraints and less time may be two important barriers to not utilizing the available dental services. Additionally, the perceived high cost of dental treatments, the fear of dental treatments and limited time availability also pose barriers [20, 21]. HPI Scores and Hu-DBI Index scores were significantly related to OHRQoL. Poor Hu-DBI scores were associated with poor OHRQoL in individuals. A person's OHB impacts their OHRQoL. OHRQoL is a subjective measure of a "person's well-being and daily functioning according to how their oral health affects them". Oral health status impacts aspects like oral function, pain, appearance and psychosocial well-being, which affects OHRQoL [2, 22 and 23]. Low HPI scores were significantly associated with low OHIP-14 scores. Diet, smoking, alcohol consumption and oral hygiene habits have direct effects on the development of oral diseases and their impact on daily function and social well-being. It is possible to experience pain, difficulty chewing, speech problems and even depression as a result of poor oral health. A number of studies carried out in varied populations have linked poor HPI Scores with poor OHRQoL [24, 25, 26]. As a cross-sectional study, the study has limitations. This study included nursing students from a single district. The population was not homogeneous because nursing students were mostly female. The self-reporting method was employed to collect data that could have biased our results. There may be other confounding factors that might affect the OHRQoL, which might not have been considered in the present study. Furthermore, OHRQoL measures are subjective and can vary among individuals with similar objective conditions.

## Conclusion:

A majority of nursing students demonstrated good Hu-DBI Scores and a good OHRQoL but, on the contrary, very few nursing students demonstrated good HPI Scores. Those with poor Hu-DBI Scores and HPI Scores had significantly worse OHRQoL. Education camps and motivational campaigns are needed to keep nursing students on track with regular dental visits.

## Figures and Tables

**Table 1:** Socio-demographic characteristics and previous dental visit of nursing students

**Factor**	**Categories**	**Number (%)**
Mean age (Mean±SD)		22.10±1.84
Location	Urban	132 (33.0%)
	Rural	268 (67.0%)
Method of cleaning	Toothbrush	333 (83.3%)
	Finger/Others	67 (16.7%)
Material used for cleaning	Toothpaste	349 (87.3%)
	Toothpowder/Others	51 (12.7%)
Frequency of cleaning	Once daily	273(68.3%)
	Twice daily	127 (31.7%)
Previous dental visit	Yes	97 (24.3%)
	No	303 (75.7%)
Socioeconomic status	Upper class	0 (0%)
	Middle class	129 (32.3%)
	Lower class	271 (67.7%)

**Table 2:** Distribution of nursing students based upon Hu-DBI Scores, HPI Scores and OHRQoL

**Factor**	**Categories**	**Number (%)**
Hu-DBI Scores	Poor	118 (29.5%)
	Good	282 (70.5%)
HPI Scores	Poor	115 (28.7%)
	Intermediate	252 (63.0%)
	Good	33 (8.3%)
OHRQoL Scores	Good	302 (75.5%)
	Poor	98 (24.5%)

**Table 3:** Association of various factors with OHRQoL in nursing students

**Factor**	**Categories**	**Good OHRQoL**	**Poor OHRQoL**	**p value**
Location	Urban	102 (77.3%)	30 (22.7%)	0.622
	Rural	200 (74.6%)	68 (25.4%)	
Method of cleaning	Toothbrush	247 (74.2%)	86 (25.8%)	0.213
	Finger/Others	55 (82.1%)	12 (17.9%)	
Material used for cleaning	Toothpaste	262 (75.1%)	87 (24.9%)	0.728
	Toothpowder/Others	40 (78.4%)	11 (21.6%)	
Frequency of cleaning	Once daily	212 (77.7%)	61 (22.3%)	0.169
	Twice daily	90 (70.9%)	37 (29.1%)	
Previous dental visit	Yes	69 (71.1%)	28 (28.9%)	0.278
	No	233(76.9%)	70 (23.1%)	
Socioeconomic status	Middle class	98 (76.0%)	31 (24.0%)	0.902
	Lower class	204(75.3%)	67 (24.7%)	
Hu-DBI Score	Poor	78 (66.1%)	40 (33.9%)	0.007*
	Good	224 (79.4%)	58 (20.6%)	
HPI Score	Poor	77 (67.0%)	38 (33.0%)	0.041*
	Intermediate	199 (79.0%)	53 (21.0%)	
	Good	26 (78.8%)	7 (21.2%)	
*p value<0.05: statistical
significant difference

**Table 4:** Multivariate Logistic regression analysis showing adjusted Odds ratio with 95% Confidence intervals for poor OHRQoL

**Factor**	**Categories**	**Adjusted Odds Ratio**	**95%CI**	**p value**
Hu-DBI Score	Poor	2.057	0.696-6.077	0.001*
	Good	Ref		
HPI Score	Poor	2.174	1.380-3.622	0.043*
	Intermediate	1.228	0.466-2.230	0.181
	Good	Ref		
*p value<0.05: statistical
significant difference
